# Identification and Mapping of Human Lymph Node Stromal Cell Subsets by Combining Single‐Cell RNA Sequencing with Spatial Transcriptomics

**DOI:** 10.1002/eji.202451218

**Published:** 2025-06-11

**Authors:** Cristoforo Grasso, Janna E. G. Roet, Catarina Gago de Graça, Johanna F. Semmelink, Michael de Kok, Ester Remmerswaal, Aldo Jongejan, Perry D. Moerland, Reina E. Mebius, Lisa G. M. van Baarsen

**Affiliations:** ^1^ Department of Rheumatology and Clinical Immunology, Amsterdam UMC, location AMC University of Amsterdam Amsterdam the Netherlands; ^2^ Amsterdam Rheumatology and Immunology Center (ARC) Amsterdam the Netherlands; ^3^ Department of Experimental Immunology, Amsterdam UMC, location AMC University of Amsterdam Amsterdam the Netherlands; ^4^ Amsterdam Institute for Immunology and Infectious Diseases Amsterdam the Netherlands; ^5^ Department of Molecular Cell Biology and Immunology, Amsterdam UMC, location VUMC VU University of Amsterdam Amsterdam the Netherlands; ^6^ Department of Epidemiology and Data Science Amsterdam UMC, location AMC University of Amsterdam Amsterdam the Netherlands; ^7^ Amsterdam Public Health, Methodology Amsterdam Amsterdam the Netherlands

**Keywords:** fibroblast, human, lymph node stromal cells, single‐cell RNA sequencing, spatial transcriptomics, stroma‐immune system

## Abstract

Lymph node stromal cells (LNSCs) have a crucial immunomodulatory function, but their heterogeneity in humans is incompletely understood. Here, we report the single‐cell RNA sequencing (scRNA‐seq) of 9267 LNSCs isolated from a human lymph node (LN). This study comprehensively defines the gene signatures of 10 fibroblast subtypes: CCL21^+^ SC, CCL19^+^ SC, CD34^+^CXCL14^+^ SC, pericytes, DES^+^ SC, LAMP5^+^ SC, NR4A1^+^BCAM^+^ SC, HLA‐DR^+^ SC, SEPT4^+^ SC and GLDN^+^ SC. The existence of these subtypes was validated across 13 LN donors using 2 publicly available datasets and our dataset. To explore the heterogeneous stromal compartment within the complex LN tissue architecture, we integrated the scRNA‐seq profiles of the identified LNSC subsets with a publicly available human spatial transcriptomic LN dataset and predicted their location within the complex LN tissue architecture. Each LNSC subtype was spatially restricted to specific LN regions, indicating different LNSC–lymphocyte interactions, which were further investigated using NicheNet. The positioning of distinct LNSC subtypes within different LN regions sets the stage for future research on the relationship between LNSC‐specific niches and immunomodulatory function during health and disease.

## Graphical abstract

Single‐cell RNA sequencing of enzymatically dissociated human lymph node stromal cells (LNSCs) reveals transcriptional and spatial heterogeneity. Following cell sorting and phenotyping, stromal subsets were validated across multiple datasets, mapped within lymph node architecture, and analyzed for subset‐specific immune interactions, enabling a comprehensive reconstruction of the LNSC landscape and its role in lymph node organization and function.

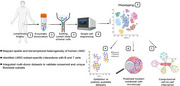

**Abbreviations**
BEC: blood endothelial cell
Cap BEC: capillary BEC
DEG: differentially expressed gene
DN: double negative
FACS: fluorescence‐activated cell sorting
FDC: follicular dendritic cell
FRC: fibroblast reticular cell
GO: gene ontology
HEV: high endothelial venous
LEC: lymphatic endothelial cell
LNSC: lymph node stromal cell
MRC: marginal reticular cell
PCA: principal component analysis
SC: stromal cell
scRNA‐seq: single‐cell RNA‐sequencing
Th: T helper cell
TRCT: zone Reticular Cell
UMAP: uniform manifold approximation and projection

## 1 Introduction

Adaptive immune responses are initiated in lymphoid organs, where immune cells surveil to protect our body against pathogens or tumor progression [1]. In a lymph node, nonhematopoietic (CD45−) and hematopoietic cells (CD45+) coexist to orchestrate immunity. The nonhematopoietic cells make up approximately 5% of the total cells of a lymph node [2]. These nonhematopoietic cells, also known as lymph node stromal cells (LNSCs) provide structural and mechanic support to the lymph node but also modulate immune cell maturation, migration, and activation [3]. Two major populations of LNSCs can be distinguished, namely endothelial (CD31+) cells, further subdivided into blood (BECs) and lymphatic endothelial cells (LECs), and nonendothelial (CD31−) cells, which are subdivided into fibroblast reticular cells (FRCs) and double negative (DN) cells [4, 5, 6, 7, 8]. Lymphocytes can access the lymph node from the blood via high endothelial cells, which form venules and support the multistep leukocyte extravasation cascade [9]. Lymphatic vessels carry self‐ and non‐self‐antigens and multiple types of immune cells to the draining lymph node, for initiating immune activation or induction of peripheral tolerance [10]. FRCs secrete components of the reticular network and regulate the traffic of lymphocytes and dendritic cells (DCs) over the network [11]. Additionally, LNSCs can present antigens [12, 13] and attract lymphocytes [14, 15] and B cells [16] thereby mediating peripheral immune tolerance [17, 18]. Technological advances, such as single‐cell RNA sequencing (scRNA‐seq) have expanded our understanding of LNSC heterogeneity [5, 19–24], and how heterogeneity may impact LNSCs’ immune modulation functions [25]. The heterogeneity of LNSCs reflects the variety of interactions and synergy with the immune system within secondary lymphoid organs. Nowadays, it is clear that dysfunction of and depletion of human LNSCs, as well as their depletion in mouse models, result in a severely impaired immune response [5, 8, 26, 27, 28]. Therefore, it is of paramount importance to investigate the role of human stromal cells (SCs) in their LN microenvironment as this may lead to the discovery of potential processes for immunomodulation. The current study employs scRNA‐seq and spatial transcriptomics to assess the heterogeneity of human LNSCs, and to identify in which areas of the lymph node each subset is located. With our approach, we found ten clusters of fibroblastic cells: NR4A1+BCAM+ SC, CCL21+ SC, CCL19+ SC, CD34+CXCL14+ SC, pericytes, DES+ SC, LAMP5+ SC, HLA‐DR+ SC, SEPT4+ SC, and GLDN+ SC. We validated the presence of these LNSC subtypes across 13 lymph node donors integrating our data with two publicly available datasets [5, 8]. Next, we predicted the locations of each subset in the lymph node by integrating our single‐cell RNA profiles with publicly available spatial transcriptomics data. Immunofluorescence staining on lymph node sections was used to validate the predicted location of the newly discovered GLDN+ SCs and CD34+ CXCL14+SCs in lymph node tissue. Furthermore, NicheNet was applied to reveal potential T‐ and B‐cell interactions with various identified LNSC subtypes.

## 2 Results

### 2.1 Single‐Cell RNA Sequencing Analysis of Human Lymph Node Stromal Cells

**FIGURE 1:**
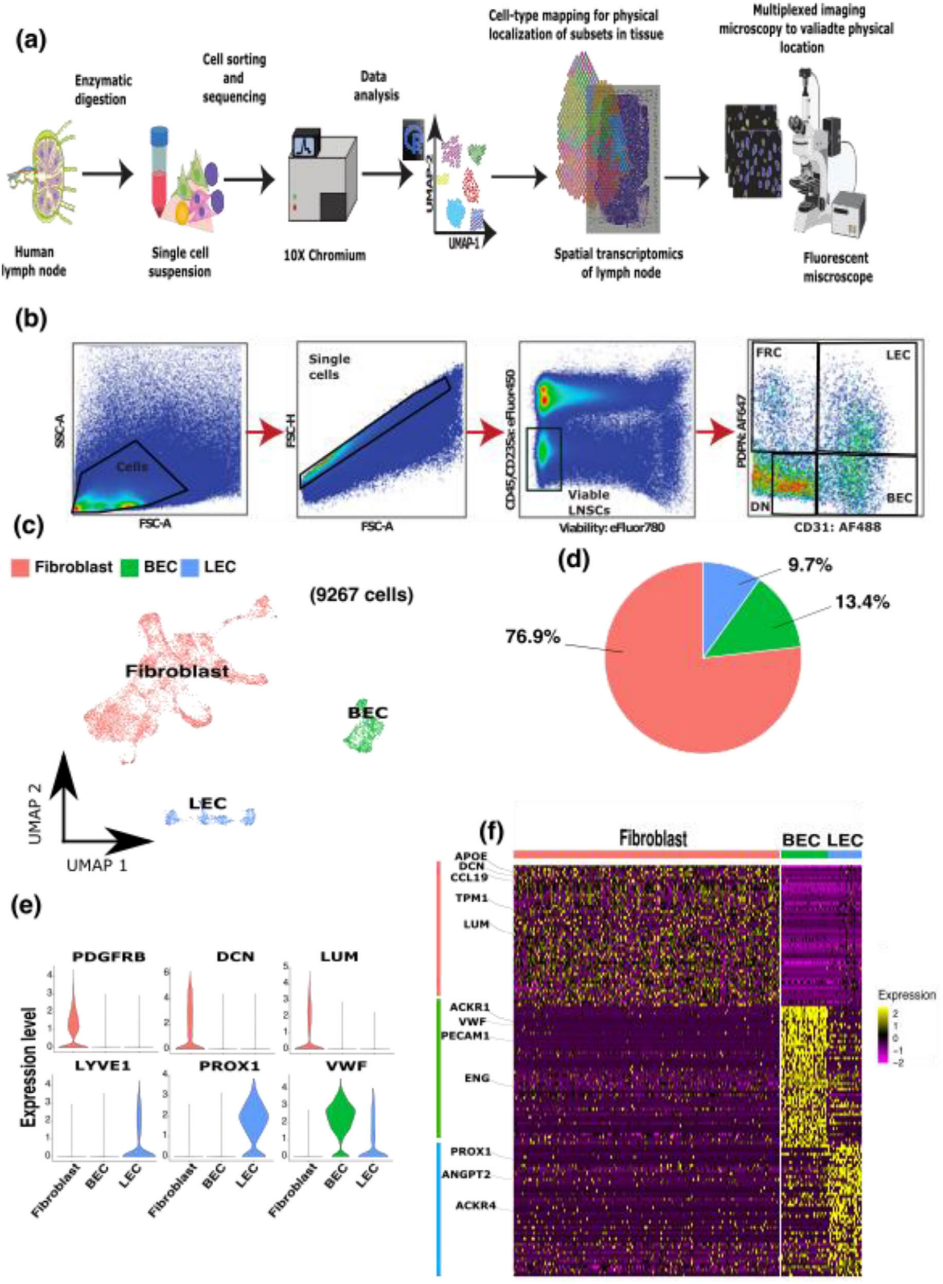
Comprehensive single‐cell RNA sequencing strategy to characterize major lymph node stromal cell (LNSC) populations. (a) Overview of the experimental pipeline used in this study. The process begins with the enzymatic digestion of a human lymph node to generate a single‐cell suspension, which is then subjected to cell sorting and sequencing using the 10× Chromium platform. The resulting data is analyzed to produce a UMAP (Uniform Manifold Approximation and Projection) representation, which clusters the cells based on their gene expression profiles. These clusters are then mapped back to their spatial locations within the lymph node tissue using spatial transcriptomics, providing a physical localization of the different fibroblast subsets. Finally, multiplexed imaging microscopy is used to validate the physical location of these subsets within the lymph node using a fluorescent microscope. (b) Flow cytometry gating strategy used to sort viable CD45‐ CD235a‐ single cells containing DN, FRC, LEC, and BEC (see Figure S1 for more details). (c) Two‐dimensional clustering of 9267 LNSCs analyzed by scRNA‐seq. LNSCs are color‐coded based on their main phenotype: fibroblasts (red), LEC (blue), and BEC (green). (d) Pie chart illustrating the proportional distribution of each LNSC phenotype among the total 9267 cells analyzed. This chart provides a quantitative overview of the relative abundance of fibroblasts, LEC, and BEC in the sample. (e) Violin plots showing the log‐normalized expression levels of marker genes specific to each LNSC phenotype. For endothelial cells (BEC and LEC), PECAM1 and VWF are used as markers; for fibroblasts (FRC), DCN, LUM, and PDGFRB are shown; and for LEC, PROX1 and LYVE1 are the defining marker. (f) Heatmap depicting the top 50 differentially expressed genes across the three main LNSC populations at the single‐cell level. Marker genes are highlighted within the heatmap, and the bars at the top of the heatmap correspond to the different LNSC phenotypes (red for fibroblasts, blue for LEC, and green for BEC). Z‐scores are shown to indicate fold‐change expression relative to the mean expression for each gene.

To reveal the heterogeneity and molecular blueprint of human LNSCs we enzymatically digested a human lymph node to obtain a single cell suspension. Subsequently, four major human peripheral lymph node CD45− cell subtypes were sorted and processed for droplet‐based single‐cell RNA sequencing (scRNA‐seq) (Figure 1A, a detailed report of our sorting strategy is included in Figure S1A). Endothelial and nonendothelial LNSCs were classified as CD45‐CD235a‐ and further divided into the four major LNSC subtypes based on the expression of PDPN and CD31: FRCs (PDPN+, CD31−), DNs (PDPN−, CD31−), LECs (PDPN+, CD31+), and BECs (PDPN−, CD31+). Accordingly, viable LNSCs were enriched by fluorescence‐activated cell sorting (FACS), while ensuring that our isolated stromal cell suspension contained cells from all major LNSC subtypes (Figure 1B). Sorted cells were sequenced with high coverage using the 10× Chromium protocol. After data preprocessing, we performed principal component analysis (PCA) followed by unsupervised clustering visualized on a UMAP plot for visualization of 9267 cells clustered using a graph‐based method. In the UMAP plot, three distinct cell types (fibroblasts, BECs, LECs) can be clearly distinguished (Figure 1C). Based on the expression of canonical markers [17, 25, 26] we annotated 7081 fibroblasts expressing *DCN*, LUM, and *PDGFRB* representing the largest group of our dataset (Figure 1D), 1’,244 BECs positive for *VWF* and 901 LECs expressing *LYVE1* and/or *PROX1* (Figure 1E). Additionally, we confirmed the annotation using differential expression analysis, which revealed a clear signature of the fibroblast, lymphatic, and blood endothelial subsets; the top 50 differentially expressed genes (DEGs) are represented in the heatmap (Figure 1F; Supporting Information Table 1). In summary, our scRNA‐seq analysis confirmed the presence of LECs, BECs, and fibroblasts within our sorted human LNSC sample. We next sought to identify specific subsets within each of these three distinct cell types.

**FIGURE 2:**
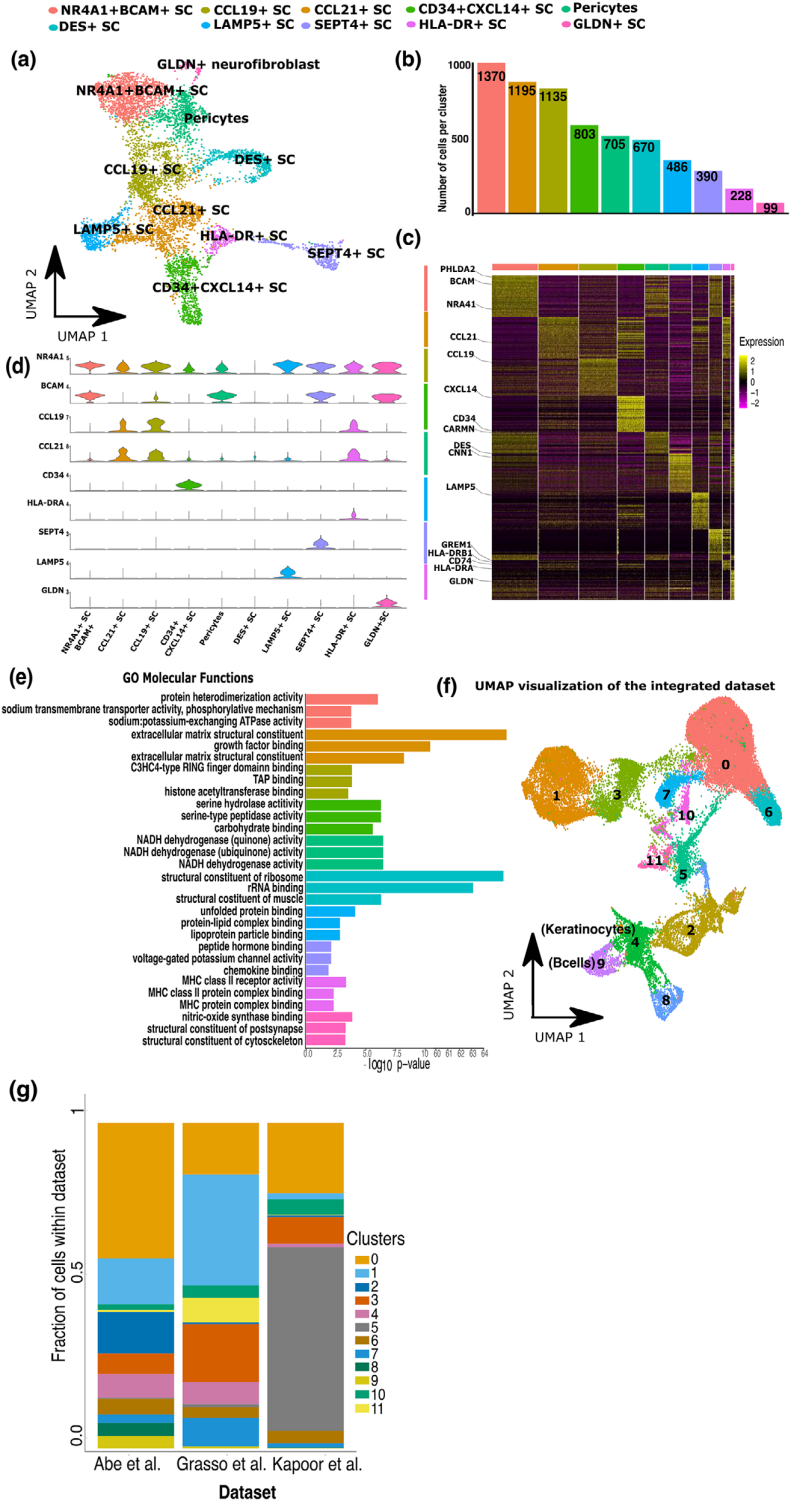
Identification and characterization of 10 lymph node fibroblast subsets via single‐cell RNA sequencing. (a) UMAP (Uniform Manifold Approximation and Projection) analysis depicting 10 distinct clusters of lymph node fibroblasts. Each cluster represents a subset of fibroblasts with a unique transcriptional profile. Cells are color‐coded according to the cluster to which they belong. (b) Bar plot displaying the absolute number of cells within each of the 10 fibroblast clusters. Each bar is colored to correspond with the UMAP clusters, providing a quantitative overview of the relative abundance of each fibroblast subset. (c) Heatmap showing per cell (column) the expression patterns of the top 50 DEGs (rows) across the 10 fibroblast clusters. Each column represents a single cell, while rows correspond to specific DEGs. Marker genes defining each fibroblast cluster are highlighted on the left, and the Z‐score normalized expression levels are indicated by the color legend on the right. The top of the heatmap includes color‐coded bars representing the assigned cluster for each fibroblast. (d) Violin plots showing Z‐score expression levels of key marker genes for each fibroblast cluster. (e) Gene Ontology (GO) enrichment analysis was conducted on the DEGs identified in each fibroblast cluster. The analysis highlights the top three GO molecular functions for each subset. (f) Integrated scRNA‐seq analysis of LN fibroblasts by combining our dataset with two publicly available datasets of Abe et al. [8] and Kapoor et al. [5]. UMAP visualization illustrates the distribution of cell subsets across the integrated dataset, comprising 42,073 lymph node cells isolated from a total of 13 donors. The integrated analysis reveals the consistency of the fibroblast clusters across different datasets. (g) This stacked bar plot shows the percentage distribution of cell clusters within three integrated datasets: Abe et al. [8], Grasso et al., and Kapoor et al. [5]. Each bar represents one dataset (*x*‐axis), and the different colors within each bar correspond to specific clusters (0–11), as indicated in the legend on the right. The *y*‐axis represents the fraction of cells within each dataset, ranging from 0.0 to 1.0, with 1.0 indicating 100% of cells within a dataset.

First, we elucidated the heterogeneity of the LEC and BEC populations. We assigned names to each subset based on a selection of key genes that were highly differentially expressed in each subset. The analysis of the LECs uncovered four major subsets: ACKR4+ LECs, ACKR1+ LECs, ANGPT2+ LECs, and CD24+ LECs (Figure S2). Unsupervised clustering analysis of the BECs also discovered four subsets, namely Cap BEC, c‐aHEV, venous BECs, and arterial BECs (Figure S3). We provide a detailed description of the LECs and BECs subsets in the Supporting Information. Overall, the identification of these LN endothelial subsets is in line with earlier reports [7, 20, 24, 27, 28]. Next, we focused on the fibroblast population in which clustering analysis identified 10 fibroblast subsets illustrated in the UMAP plot (Figure 2A). The number of cells per subset is shown in Figure 2B. Additionally, a variety of DEGs were identified in each subset (Supporting Information Table 2). The top 50 DEGs for each fibroblast subset showing the highest fold change (log2‐fold change >0.5, adj. *p*‐value < 0.05, proportion of cluster expressing > 0.25) are visualized in a heatmap with key genes highlighted for each subset (Figure 2C). For reference, we assigned a name to each subset based on a selection of key genes highly differentially expressed in each subset (Figure 2D). Subsequently, the DEGs were used as input for a gene ontology (GO) enrichment analysis reflecting functional differences between each fibroblast cluster subset. This analysis indicated a unique role for each subset crucial for the functionality of a lymph node (Figure 2E).

To ensure the robustness and replicability of our findings, we integrated our data with two publicly available human datasets from Abe et al. [8] containing fibroblasts from nine lymph nodes and Kapoor et al. [5] containing fibroblasts from three lymph nodes. This analysis suggests that the gene expression signatures of our identified LNSC subsets could be largely replicated in the other two datasets (Figure 2F,G). However, it is important to note the presence of a few highly dataset‐specific clusters, namely clusters 0, 2, and 8 (Abe et al. [8]) and cluster 5 (Kapoor et al. [5]) indicative of variability inherent to the respective datasets. These clusters highlight the methodological (e.g., tissue processing) and biological differences (e.g., donor and tissue origin) that can emerge between datasets. The distribution of clusters across datasets (Figure 2G) and the relative contributions of each dataset per identified cluster (Figure S4A) reflect the alignment of the different datasets and support the existence of our identified LNSC subsets while also highlighting dataset‐specific variations. These findings may reflect methodological differences as well as potential biological differences between donor samples. To further explore the identified fibroblast LNSC subsets within the integrated dataset, we utilized the AddModuleScore function in Seurat, using the differentially expressed genes from each of the ten fibroblast subsets as a signature.

This analysis, visualized in a heatmap (Figure S4B), revealed that certain subsets had high module scores for specific clusters, indicative of a distinct presence within the integrated dataset. For instance, CCL19+ SC has the highest score in cluster 3, CCL21+ SC in cluster 10, LAMP5+ SC in cluster 7, and CD34+CXCL14+SC in cluster 6. Notably, the relatively large cluster 1 was identified for targeted re‐analysis due to its high scores for several LNSC modules. Re‐clustering of cluster 1 separately resulted in clearer discrimination of the different LN fibroblast subtypes. This additional analysis reinforces the distinctiveness of these subtypes and confirms their presence across datasets (Figure S4C,D). As anticipated, module scores were notably low for the clusters within the integrated dataset unlikely to consist of LN fibroblasts, such as cluster 9 (B cells) highly expressing MZB1 (82.5% of the cells in the cluster expressing the gene, adjusted *p*‐value = 0), cluster 4 (keratinocytes) highly expressing KRT18 (73.7% of cells, adjusted *p*‐value = 2.6E‐189), KRT19 (73% of cells, adjusted *p*‐value = 0), and KLK1 (87.3% of cells, adjusted *p*‐value = 0), and dataset‐specific clusters 0, 8, 2 (Abe‐specific) and 5 (Kapoor‐specific) (Figure 2F; Figure S4B; Supporting Information Table 3). These lower module scores reflect an additional specificity control, affirming the distinct expression profiles of our identified LN fibroblast subtypes and negating dataset‐specific noise influences.

In summary, this explorative study on a relatively large number of sorted human LNSCs unveiled the presence of 10 subsets of fibroblasts in human LN tissue, of which some have been earlier described, for example, CCL19+ SC, CCL21+ SC, CD34+ CXCL14+ SC, DES+ SC, NR4A1+ BCAM+ SC, and pericytes, whereas others are newly discovered, e.g., SEPT4+ SC, GLDN+ SC, LAMP5+ SC, and HLA‐DR+ SC. Below we further investigate the spatial location and functionality of each identified LN fibroblast subset.

### 2.2 Spatial Distribution of Lymph Node Fibroblast Subsets

**FIGURE 3:**
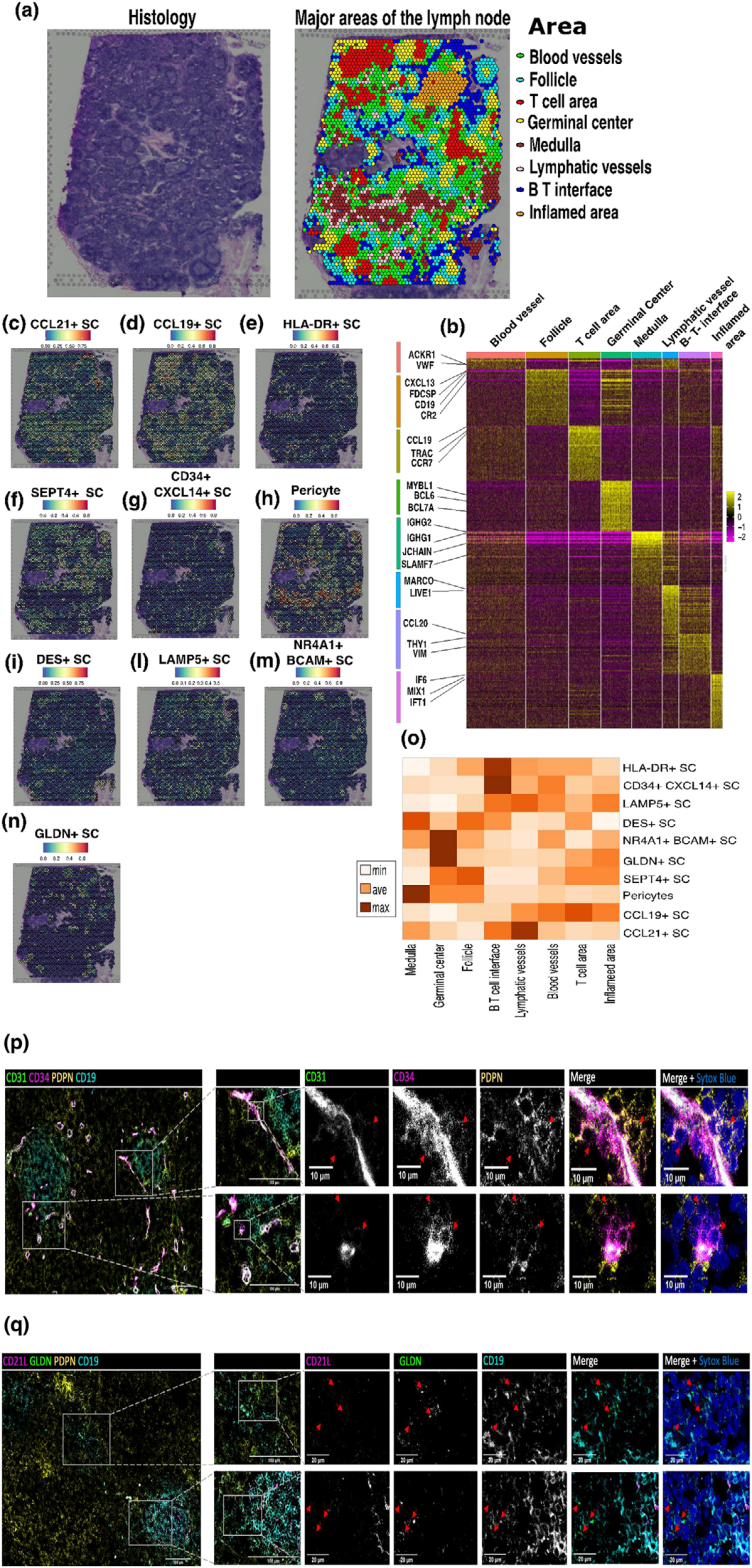
Spatial distribution of fibroblast subsets within the lymph node revealed by spatial transcriptomics and microscopy. (a) On the left, a histological section of a human lymph node stained with hematoxylin and eosin (H&E), highlighting the structural components of the lymph node, such as follicles, germinal centers, and medullary areas. The image is adapted from 10× Genomics. On the right, spatial gene expression profiles corresponding to the same histological section, reveal distinct regions such as germinal centers, follicles, T‐ and B‐cell areas, medulla, blood vessels, and lymphatic vessels. Each region is color‐coded (see legend) to illustrate its spatial distribution (see also Figure S6A,B for more details on the analysis). (b) Heatmap showing the differentially expressed genes (DEGs) used to define and annotate the major regions of the lymph node. The color‐coded bars correspond to each region, with Z‐scores on the right representing the relative gene expression levels across these regions. (C–N) Spatial maps displaying in‐silico prediction scores for the presence of each fibroblast subset at various locations within the lymph node. Prediction scores range from 0 (blue, indicating no predicted presence) to 1 (red, indicating a high likelihood of presence). (O) Two‐dimensional heatmap showing the clustering of mean prediction scores per area of the lymph node for each fibroblast subset. The mean prediction values were grouped into low, medium, and high scores, indicating the likely abundance of each fibroblast subset across different regions of the lymph node. (P) Composite immunofluorescence micrographs (acquired at 40× magnification) of a human lymph node section. Specific markers, including CD31 (green), CD34 (magenta), PDPN (yellow), and CD19 (cyan), are displayed in individual panels, and the “Merge” panel combines the staining patterns of CD31, CD34, and PDPN to reveal co‐localization. The “Merge + Sytox Blue” panel adds a nuclear counterstain. Inserts in the micrographs magnify regions of particular interest, with arrows indicating co‐localization of specific markers. Scale bars are 20 µm for individual panels and inserts, and 100 µm for the full section. Representative images from one of three different lymph nodes are shown. (Q) Immunofluorescence staining of another human lymph node section, (acquired at 40× magnification), showing the distribution of markers CD21L (magenta), GLDN (green), PDPN (yellow), and CD19 (cyan) within the tissue architecture. The full section (leftmost panel) illustrates the spatial localization of these markers. The upper and lower rows of magnified inserts focus on two distinct areas of interest within the lymph node. Individual panels provide detailed views of each marker, while the final panel merges all the markers along with Sytox Blue as a nuclear counterstain. Scale bars are 20 µm for individual panels and inserts, and 100 µm for the full section. Representative images from one of three different lymph nodes are shown.

We explored the spatial distribution of identified fibroblast subtypes within the lymph node by transferring cell type annotations from our scRNA‐seq data onto a publicly available spatial transcriptomic LN dataset [29]. This provided prediction scores indicating the presence of specific cell types in defined regions of the lymph node [29, 30]. First, we performed cluster analysis on the spatial data and visualized the results using a UMAP plot (Figure S5A). Second, we plotted the cluster identity of each spot of the spatial data and confirmed that the identified clusters are spatially restricted to specific LN regions as depicted in the histological section (Figure 3A, left panel). The histology provides a microscopic view of the lymph node's structure, which correlates with the spatial distribution of the spatial transcriptomics gene clusters (Figure 3A, right panel). Next, we manually annotated each spatial spot based on the well‐established function of genes differentially expressed between specific areas of the lymph node (Figure 3B; Figure S5B; Supporting Information Table 4). Through this analysis, we identified distinct spatial domains such as T‐cell areas (expressing TRBC1, TRAC), follicles (expressing FDCSP, CR2), germinal centers (expressing BCL6, MYBL1), the B–T cell interface (expressing THY1), medulla (expressing IGHG1, IGHG2), lymphatic vessels (expressing LYVE1, PROX1), and blood vessels (expressing VWF, PECAM1). We then integrated our scRNA‐seq dataset with the spatial transcriptomic dataset to obtain for each of the discovered LN fibroblast subsets an in silico prediction score for localization in each spot of the lymph node tissue spatial transcriptomic dataset (Figure 3C–N). To depict the predicted locations of these subsets, we calculated the mean prediction value for each lymph node region and visualized these in a heatmap (Figure 3O). This heatmap highlights, for example, that the medulla region, rich in medullary cords, is enriched with pericytes and DES+ SCs that are known to surround vessel‐like structures. In contrast, the newly identified GLDN+ SC was specifically predicted to be located within the germinal center regions of the lymph node. Our spatial analysis predicts that each identified LN fibroblast subset occupies a different niche within the lymph node, which suggests that each LNSC subset also harbors a niche‐specific function within the LN. In the following sections, we speculate on the role of these subsets based on their DEGs, predicted locations, gene ontology analysis, and possible interactions with other cells co‐localized within the same region of the lymph node.

#### 2.2.1 CCL19+ and CCL21+ SC

Starting from the cortex of the lymph node, CCL19+ SCs also called T‐zone reticular cells (TRCs) [19] expressed high levels of CC chemokine ligand 19 (*CCL19*), which guides CCR7+ T cells to the paracortex of the lymph node [31]. In our dataset, CCL19+ SCs express high levels of *CCL2* in the vessel‐rich areas of the T‐cell area where they might attract monocytes, as previously described for FRCs dwelling in the T‐cell area [32]. Furthermore, it is worth noting that LNSCs predominantly rely on fatty acid metabolism [33]. This is evident through the high expression in CCL19+ SCs of CD36, FABP4, and FABP5, proteins that play crucial roles in the fatty acid metabolism [34], *FABP4* and *FABP5* also are involved in the maintenance of T lymphocyte homeostasis through the modulation of cytokine production in thymic stromal cells [35]. Additionally, the T‐cell area is populated by the CCL21+ SCs, which highly express *CCL21* and are further characterized by lower *CCL19* expression compared with CCL19+ SCs. *CCL21* is a chemokine involved in the attraction and homing of CCR7+ T‐cells [36]. CCL21+ SCs also highly express stromal cell‐derived factor 1 (CXCL12), known for attracting CXCR4+ lymphocytes and monocytes to the lymph node. The gene expression profiles of CCL19+ SCs and CCL21+ SCs support the mapping analysis, which indicated that CCL19+ LNSCs are mostly located in the inner cortex of the T‐cell area whereas CCL21+ LNSCs are predicted to be located more in the outer cortex, closely to the B–T cell interface and in the proximity of lymphatic vessels (Figure 3C,D,O).

#### 2.2.2 HLA‐DR+ SCs and SEPT4+ SCs

Our data indicates that HLA‐DR+ SCs uniquely express high levels of *HLA‐DRB1*, *HLA‐DRA*, and *CD74*, which are components of the HLA class II antigen presentation machinery [37]. Previous human studies have indeed reported the expression of HLA class II molecules on human LNSCs [13, 38, 39]. Furthermore, murine MHC‐II+ FRCs have been found to be important in regulating antigen‐specific CD4+ T cell responses [13, 40]. *GREM1* was also highly expressed by HLA‐DR+ SC. GREM1+ SCs have been recently described as FRCs providing a niche for CD4+ T cells in the B–T cell interface of the lymph node and involved in interaction with DCs [5]. Our spatial mapping analysis indeed positioned HLA‐DR+ SCs in the B‐T cell interface (Figure 3E,O). Based on their transcriptome profile and their predicted location within the lymph node, human HLA‐DR+ SC may engage in a self‐antigen presentation to CD4+ T cells like their murine counterparts [13, 18]. SEPT4+ SCs remarkably express several genes of the septin family including *SEPT7*, *SEPT2*, *SEPT4*, *SEPT9*, *SEPT10*, and SEPT11 [41]. Septins, in collaboration with actin, organize the filamentous network of the cellular cytoskeleton in dividing and not dividing cells [42, 43], and contribute to autophagosome biogenesis [44]. Our spatial analysis predicts that SEPT4+ SCs are mostly located in the follicles (Figure 3F,O). We observed that HLA‐DR+ SCs and SEPT4+ SCs both highly express septin genes (Supporting Information Table 2) but differ in the expression of genes associated with FRCs such as *LUM*, *DCN*, and *GREM1*. These findings and the UMAP plot (Figure 2A) suggest that SEPT4+ SCs are functionally related to HLA‐DR+ SCs; however, further investigation is needed to confirm this.

#### 2.2.3 CD34+CXCL14+ SCs

In the adventitia of the vasculature CD34+ SCs may act as progenitor cells that give rise to T zone reticular cells (TRCs), follicular dendritic cells (FDCs), marginal reticular cells (MRCs) and pericytes [45, 46]. As previously reported in mice, CD34+ SCs highly express collagens *COL3A1*, *COL6A2*, and *COL1A*2 [46]. Moreover, in our dataset and the integrated dataset CD34+ CXCL14+ SCs highly co‐express PI16 and CD34 (Figure S6). Recently, PI16 has been reported as a marker for “universal” progenitors of fibroblast lineages [47, 48, 49]. Our mapping analysis assigns high scores for CD34+CXCL14+ SCs in the proximity of blood vessels, but surprisingly even higher scores for predicted location in the B–T‐cell interface area (Figure 3G,O). In human lymph node sections, immunofluorescence microscopy revealed the presence of CD34+PDPN+CD31– stromal cells in the proximity of blood vessels that were expressing both CD34 and CD31 (Figure 3P). Aligned with the spatial mapping, the images show the presence of CD34+PDPN+CD31– stromal cells surrounding the CD34+CD31+ blood vessels in the B–T cell interface, as marked by the magnified inset in Figure 3P (top row), which is consistent with previously described mouse studies [19, 50]. The transcriptional profile and predicted location of human CD34+ CXCL14+ SCs suggest that this subset indeed may contain progenitor cells giving rise to other lymph node fibroblast subsets, but this warrants further investigation.

#### 2.2.4 Mural Stromal Cells

The identified pericytes, DES+ SC, and LAMP5+ SC seem to mural cells surrounding vessel‐like structures within the lymph node. Accordingly, we observed that pericytes and DES+ SCs have high prediction scores in the medulla (Figure 3H,I,O), which is rich in medullary cords, whereas LAMP5+ SCs are spatially close to lymphatic and blood vessels (Figure 3L,O). Pericytes regulate and support the microvasculature through direct contact with the endothelium [51, 52]. In our dataset pericytes express *MCAM*, *CARMN*, and *NET1* consistent with recent studies where human pericytes have been analyzed [53, 54, 55]. In our dataset, they also express *RERGL* and *RGS5*, which have been described as markers for both pericytes and smooth muscle cells in human skin and ovarian cortex [56, 57]. Previous studies have described a population of DES+ smooth muscle cells closely wrapping human small muscular arteries [58] and LNSCs expressing desmin with muscle‐like features in human lymph nodes [59, 60]. In line with this, our identified DES+ SCs highly express genes known to be expressed in smooth muscle cells, including *CNN1, MYL6, MYL9, MYLK, TPM1*, and *TMP2* [61, 62]. In our dataset, LAMP5+ SCs highly express a variety of genes known to modulate blood pressure and vasculogenesis of the blood vasculature, such as *AGT*, *F5*, and *SFPR* [63, 64]. Overall, our study confirms that the lymph node pericytes and DES+ SCs may co‐operate with endothelial cells as previously described [19, 65, 66]. Also, these pioneering findings show that gene expression profiles and spatial mapping suggest that LAMP5+ SCs may be closely associated with the endothelial cells lining the lymph node vasculature. However further studies are required to investigate the precise role of LAMP5+ SCs.

#### 2.2.5 NR4A1+ BCAM+ SCs

NRA41+ BCAM+ SCs are predicted to reside in germinal centers (Figure 3M,O). As previously described in mouse [19], also in our dataset NR4A1+ BCAM+ SCs highly express *NR4A1*, *BCAM*, *FOSB*, *FOS*, *JUNB*, *EGR1*, *NFKBIA*, and *ZFP36*. In mice, this subset is considered to consist of a mixture of different activated cells from other LNSC subsets [19]. We found in our dataset that NR4A1+ BCAM+ SCs highly express *BCAM* and *MCAM*, which belong to the laminin family of receptors for extracellular matrix proteins. In murine lymph nodes, FRC‐derived laminins have been described to be crucial for the localization and transmigration of Th1, Th2, and Th17 [67]. In addition, in murine germinal centers, laminin‐binding integrins expressed by lymphocytes, promote their migration [68]. The role of human NRA41+ BCAM+ SCs in immunomodulation and lymphocyte trafficking within germinal centers should be further explored to comprehend the functionality of this subset.

#### 2.2.6 GLDN+ SC

Next to genes typically expressed by stromal cells, GLDN+ SC highly expresses genes related to neural cells such as *GLDN*, *PCDH10*, *KCNMA1*, ALKAL2, and *ADGRL3* [69, 70, 71, 72, 73, 74]. *GLDN* and *ADGRL3* are involved in the localization of the node of Ranvier and neuron guidance [69]. *KCNMA1* encodes the voltage‐ and calcium‐activated potassium channel [75] and *PCDH10* is a neural receptor [76]. It has been described that in certain contexts fibroblasts can express neural markers, e.g., human dermal fibroblasts expressing *GLDN* [77] and *FBXO32* expressed by epithelial cells in the human brain during epithelial–mesenchymal transition [78]. Our mapping analysis revealed that GLDN+ SC is located within germinal centers (Figure 3N,O). We confirmed our results through immunofluorescence microscopy of human LN tissue, which was stained for PDPN, GLDN, and CD19 as a marker for B cells, the long isoform of CD21 (CD21L) to delineate FDCs, and neurofilament light (NEFL), as a marker for neuronal cells. Our analysis showed that GLDN‐positive and NEFL‐negative fibroblasts are predominantly found in the follicles, as marked by CD19 (Figure 3Q, upper and lower row). As B cell follicles are populated with FDCs we included CD21L, to determine if GLDN is expressed on FDCs. The images demonstrate that GLDN and CD21L do not co‐localize, indicating that GLDN is not expressed by FDCs (Figure 3Q, upper and lower rows). Furthermore, the absence of NEFL expression in GLDN‐positive cells confirms that they are fibroblasts rather than neurons (Figure S7A). To validate the specificity of the NEFL antibody we tested it on the human brain section (Figure S7b). Huang et al. [79] demonstrated in mice that LNs are innervated by unique neural cells that signal and highly interact with stroma, for supporting immune cell types in LNs. Accordingly, it has been reported in mouse spleen and lymph nodes that interaction between nerve fibers and lymphocytes takes place where humoral immune responses are initiated [80]. As shown in the spatial dataset in the current study, neuronal markers *TUBB* and *TUBB4* [81] are highly expressed in the follicles, suggesting a high density of neurons in this area of a human lymph node (Figure S5C). Based on the provided evidence, it will be interesting to study the interaction between GLDN+ SC and nerve cells in human lymph nodes and how this neuron–stroma axis complements the immune response.

### 2.3 Region‐Specific Fibroblast‐Immune Cell Interactions in the Lymph Node Identified by Ligand–Receptor Analysis

**FIGURE 4:**
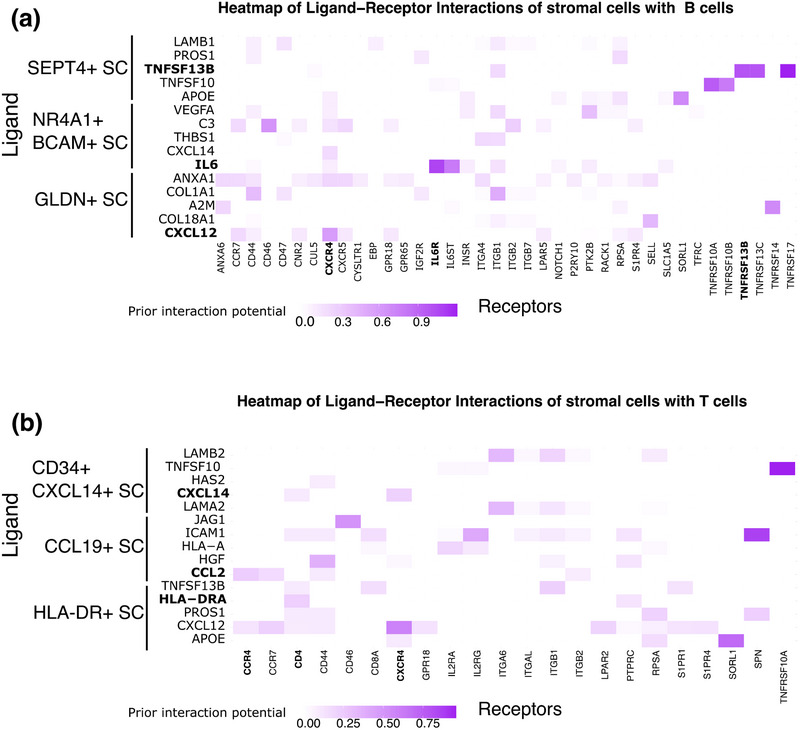
Heatmaps of ligand–receptor interactions between stromal cell subsets and B or T cells. (a) Heatmap displaying ligand–receptor interactions between three stromal cell (SC) subtypes (GLDN+ SC, SEPT4+ SC, and NR4A1+ BCAM+ SC) and B cells. The *x*‐axis represents receptor genes, and the y‐axis represents the stromal cell subtypes expressing specific ligands. Warmer colors (ranging from light to dark purple) indicate stronger interactions (higher weight scores, see values Supporting Information Table 5). (b) Heatmap showing ligand–receptor interactions between three additional stromal cell subtypes (CD34+ SC, CLL19+ SC, and HLA‐DR+ SC) and T cells. The *x*‐axis displays receptor genes, while the *y*‐axis shows stromal cell subtypes expressing specific ligands. Stronger ligand–receptor interactions are indicated by warmer colors (ranging from light to dark purple; see values Supporting Information Table 6).

Our analysis predicts that stromal cell subset cells are located in different areas of the lymph node, including areas that are populated by specific immune cells, for example, T cells in the T‐cell area and B cells in germinal centers. These data suggest that LN fibroblast subtypes can interact with certain immune cells located at specific regions throughout the lymph node. Guided by our data, we explored potential interactions between fibroblast subsets and immune cells (specifically T cells and B cells) using NicheNet, a tool for identifying and analyzing ligand–receptor interactions [82]. For this analysis, we used differentially expressed ligands of each stromal cell subset within two groupings: stroma–T cell interactions and stroma–B cell interactions. For B cells, we analyzed interactions with subsets predicted to reside in germinal centers, that is, SEPT4+ SC, NR4A1+ BCAM+ SC, and GLDN+ SC (Figure 3O). For T cells, we investigated interactions with stromal cell subsets predicted to localize to the T‐cell area and the T–B cell interface, that is, CCL19+ SC, CD34+CXCL14+SC, and HLA‐DR+ SC (Figure 3O). This approach was designed to validate known interactions (e.g., CCL19+ SC with T cells, a pairing well‐documented in the literature) while uncovering potential novel interactions (e.g., GLDN+ SC with B cells). By confirming the accuracy of predicted interactions involving well‐characterized cell pairs, we aim to lend further credibility to the identification of previously unreported interactions. Our analysis identified several novel interactions, while some interactions have been previously reported on LNSCs, such as TNFSF13B‐TNFRSF13B, IL6‐IL6R, CXCL12‐CXCR4, CXCL14‐CXCR4, and CCL2‐CCR4. For instance, we found TNFSF13B‐TNFRSF13B, IL6‐IL6R, and CXCL12‐CXCR4 interactions for the GLDN+ SC, NR4A1+ BCAM+SC, and SEPT4+ SC with B cells respectively (Figure 4A, selected ligands and receptors in bold). Similarly, we identified CXCL14‐CXCR4, CCL2‐CCR4, and HLA‐DRA‐CD4 interactions between CCL19+ SC, CD34+ CXCL14+ SC, and HLA‐DR+ SC with T cells (Figure 4B, selected ligands and receptors in bold). These findings suggest that distinct LN fibroblast subtypes may exert diverse immunomodulatory effects depending on the specific fibroblast‐immune cell pairing and that the approach provides valuable insights into the role of stromal cells in their respective niches. Importantly, novel interactions require further experimental validation to confirm their functional relevance.

## 3 Discussion

Our findings reveal a previously unpresented transcriptional, spatial, and functional heterogeneity of human LNSCs. We integrated our human single‐cell LN stromal cell data with publicly available spatial [29] and scRNA‐seq datasets from Abe et al. and Kapoor et al. [5, 8] to delineate the heterogeneity of the human LN fibroblast compartment. The integration of our dataset with those of Abe et al. [8] and Kapoor et al. [5] resulted in a dataset containing scRNA‐seq data from 13 lymph nodes and was instrumental in validating our findings, demonstrating the robustness of our identified LNSC subsets. Dataset‐specific clusters also offered insights into methodological disparities, due to cell selection biases and tissue processing techniques. For instance, the Kapoor dataset's focus on PDPN+ CD31− cells contrasts with the broader cell selection in Abe's and our datasets, which may account for observed variations in cluster composition.

Importantly, while many of these subsets share similarities across species and datasets, our integration efforts also highlight dataset‐specific variations. Beyond differences in tissue sampling and processing, these dataset‐specific variations may also reflect biological differences in microenvironmental cues, tissue‐specific adaptations, and dynamic shifts in stromal cell states influenced by immune interactions, developmental trajectories, or pathological conditions. For instance, differential exposure to cytokines, stromal‐immune interactions, and metabolic states could drive functional heterogeneity within LNSC subsets. Such factors may explain why some fibroblast populations are restricted to specific datasets.

Of note, our ligand–receptor analysis was not aimed at identifying preferred lymphocyte partners for each stromal subset but at exploring whether the predicted colocalization of stromal and immune cells could lead to ligand–receptor interactions indicative of cell–cell communication. Using NicheNet, we predicted ligand–receptor interactions between CCL19+ SC, CD34+ CXCL14+ SC, and HLA‐DR+ SC with T cells in the T‐cell area, and between SEPT4+ SC, NR4A1+ BCAM+ SC, and GLDN+ SC with B cells in germinal centers. This confirmed well‐known interactions, such as CCL19+ SC with T cells, and identified potential new interactions, such as GLDN+ SC with B cells. These results suggest that distinct LN fibroblast subtypes can interact with B or T cells through different ligand–receptor pairs, potentially leading to different immunomodulatory outcomes. In line with other studies identifying fibroblast diversity within lymph nodes, our findings contribute to a refined categorization of LNSC subsets, highlighting unique functional and spatial characteristics. For instance, subsets such as the CCL19+ SC, CCL21+ SC, NR4A1+ BCAM+ SC, CD34+ CXCL14+ SC, and pericytes, were previously described in mouse lymph node, though using a different name like Ccl19^high^ TRC, Ccl19^low^ TRC, Nr4a1+ SC, CD34+ SC and the pericytes included in the nonadventitial PvCs [19]. Similarly, CCL19+ SC and CCL21+ SC are reported in human studies under the umbrella name of TRC and pericytes. [8]. Progenitor phenotype CD34+ CXCL14+ SCs were previously reported in human single‐cell studies as CD34+ SCs or adventitial stromal cells to highly express CD34 and/or PI16 [5, 47–50]. HLA‐DR+ SC described in our study highly expresses GREM1, indicating that this subset may be like the reported GREM1+ SC in humans [5]. Ultimately, our data shows that LAMP5+ SCs highly express *AGT*, which is in line with the AGT SC described in humans [8]. A notable challenge identified through our integration efforts is the lack of uniform nomenclature for LNSC subsets across different datasets. This inconsistency highlights the critical need for a concerted effort within the field to standardize the naming and classification of LNSC subsets and to improve communication and collaboration among researchers. Although the current study is unique as it concerns nondisease human LN tissue data, there are some limitations to discuss. Further functional experiments are required to validate our findings for each LNSC subset. Even though we sequenced LN stromal cells from only one donor, we could validate our approach by confirming the location of the newly discovered GLDN+ SCs, and the CD34+ CXCL14+ SCs via microscopic analysis of independent human lymph node tissue sections. Moreover, the integration of our scRNA‐seq data with publicly available spatial transcriptomics data [29] corroborated findings that were initially identified through scRNA‐seq alone. This underlines the significance of combining spatial and scRNA‐seq data. Overall, this study provides a data‐dense blueprint of the human LNSC compartment during homeostatic conditions. Our findings indicate that the identified LNSC subsets, characterized by different gene signatures and mapping to specific locations within a healthy human lymph node, each harbor a specialized function that warrants further exploration.

## 4 Data Limitations and Perspectives

This study provides a high‐resolution map of human LNSC heterogeneity under homeostatic conditions, yet several limitations should be acknowledged. A key limitation is the single‐donor origin of our scRNA‐seq dataset, which may not fully capture donor‐to‐donor variability. To mitigate this, we integrated publicly available datasets from Abe et al. [8], Kapoor et al. [5], and 10× Genomics, incorporating lymph node data from 13 human donors. While this enhances robustness, larger and more diverse cohorts are needed to validate these findings.

Differences in cell selection and processing across datasets introduce potential biases. For instance, our dataset and Abe et al. used broad selection approaches, while Kapoor et al. focused on PDPN⁺ CD31⁻ stromal cells, influencing observed cluster compositions. Spatial validation using the 10× Genomics Visium dataset supports our findings, yet standardizing methodologies across studies will improve reproducibility. Another challenge is the inconsistency in LNSC subset nomenclature across studies. Many of our identified subsets align with known populations but have been reported under different names. A standardized classification system would facilitate cross‐study comparisons. Although our in silico validation was performed on several LN donors, and the localization of two identified LNSC subsets was confirmed using tissue microscopy, additional experimental validation is needed. Future studies should assess LNSC heterogeneity across disease contexts to determine its role in immune function and pathology.

## 5 Methods

### 5.1 Stromal Cell Preparation and Flow Cytometry Sorting

The study was conducted according to the guidelines of the Declaration of Helsinki, and approved by the Ethics Committee of the Amsterdam UMC, location AMC (protocol code 2016_045; 1 April 2016). We used a whole peritoneal LN collected from a kidney transplantation recipient during living donor kidney transplantation as described before [83]. The tissue did not show any signs of inflammation. As previously described [84], the adipose tissue and other surrounding tissue were manually removed with a scalpel. Subsequently, the lymph node was digested using the enzymatic mixture of 0.2 mg/mL collagenase P (Roche), 0.8 mg/mL Dispase II (Roche), and 0.1 mg/mL DNase I (Roche) in RPMI medium without serum (Gibco). After, cells were twice washed in PBA buffer (PBS containing 0.01% NaN3 and 0.5% bovine serum albumin (BSA). Cell suspensions were filtered through a 100‐µm nylon cell strainer (BD Falcon). Single‐cell suspension was stained for sorting flow cytometry using antibodies against CD31 (AlexaFluor488, Biolegend, Cat# 33110, clone WM‐59), HLA‐DR (PE, eBiosciences, Cat# 12‐9952‐42, clone L243), PDPN (Angiobio, Cat# 11‐009, clone NC‐08), Goat‐Anti Rat IgG (Alexafluor647, Invitrogen, Cat# A21247), CD235a (eFluor450, eBioscience, Cat# 48‐9987‐42, clone HIR2), CD45 (eFluor450, eBioscience, Cat# 48‐0459‐42, clone HI30), Viability dye (eFluor780, Invitrogen, Cat# 65–0865). The suspension was sorted at the FACSAria IIu SORP‐4 lasers (BD Bioscience). After sorting freshly sorted cells were immediately counted and processed according to 10x Genomics guidelines (CG000126 Guidelines for Optimal Sample Prep Flow Chart RevA).

### 5.2 Library Preparation for Single‐Cell mRNA‐Sequencing

The 2 sorted samples (HLA‐DR+ and HLA‐DR− LNSCs) were individually sequenced at the Amsterdam UMC genomics core facility after which data were merged. Single‐cell RNA‐sequencing libraries were prepared according to the manufacturer's instructions (CG000204 Rev D) using Chromium Next GEM Single Cell 3ʹ GEM, Library & Gel Bead Kit v3.1 (10× Genomics, PN‐1000121) and Chromium Next GEM Chip G Single Cell Kit (10× Genomics, PN‐1000120). Shortly, cells were combined with reverse transcriptase (RT) Master Mix and partitioned into nanoliter‐scale Gel Bead‐In‐EMulsions (GEMs) using 10× GemCode Technology where the poly‐A transcripts are captured and barcoded with an Illumina TruSeq Read 1 sequence, a 16 bp 10× barcode (unique for each individual cell), a 12 bp Unique Molecular Identifier (UMI; unique for each transcript). GEM generation and Barcoding including fragmentation, end repair and A‐tailing, double‐sided size selection, adapter ligation, clean‐up, index PCR, double‐sided size selection, and QC were performed according to the protocol provided by 10× Genomics (https://support.10xgenomics.com/single‐cell‐gene‐expression/library‐prep/doc/user‐guide‐chromium‐single‐cell‐3‐reagent‐kits‐user‐guide‐v31‐chemistry). Library quality control was performed with the Bioanalyzer HS DNA chip, like the quality check of the cDNA earlier in the protocol. Quantification was performed using a Qubit concentration measurement assay (Quant‐it DNA HS Assay kit, ThermoFisher, Q32854). Sequencing was performed with Illumina HiSeq4000 using 2 × 150 bp chemistry.

### 5.3 Processing Raw Data From scRNA‐Seq of 10× Genomics

Demultiplexing of raw base call (BCL) files and conversion to FASTQ files was performed with the Cell Ranger pipeline (10x Genomics, version 3.0.2) using “cellranger mkfastq”. Alignment to the human reference genome (10× Genomics; GRCh38 version 3.1.0), read filtering, barcode correction, UMI counting, and filtering of empty barcodes was performed using “cellranger count” to generate feature counts for every single cell. The two samples were combined using the function “merge” (Seurat), resulting in a dataset containing 13,259 cells. The mean number of UMI counts per cell was 47,582, and the median number of expressed genes for each cell was 1778. To remove cells of low quality, cells expressing less than 300 genes and cells with more than 20% of reads mapping to mitochondrial genes were filtered out. Genes with at least one feature count in more than three cells were used in the analysis.

### 5.4 Data Analysis of scRNA‐Seq Data

The filtered count data were analyzed using the Seurat package (version 3.2). Global scaling normalization was applied with the “NormalizeData” function via the “LogNormalize” method and a scale factor of 10,000. Next, we applied a linear transformation using “ScaleData” prior to the dimensionality reduction. Highly variable genes were identified by “FindVariableFeatures” (selection.method = “vst”) and used for principal component (PC) analysis. The first 50 PCs were used for nonlinear dimensionality reduction using UMAP and cell clustering. The resolution of “FindClusters” was set to 0.5, because the resulting clusters were consistent with the mapping of known markers. For a first pass of filtering, reference‐based annotation was performed using the “BlueprintEncodeDataset” from SingleR [85] (version 3.12). Hematopoietic cells were filtered out (B cells, CD4+ T‐cells, CD8+ T cells, HSCs, macrophages, melanocytes, monocytes, NK cells) and others such as mesangial cells, epithelial cells, chondrocytes, astrocytes, and melanocytes (Table S1) After filtering, the stromal cell dataset was trimmed using the function “DietSeurat”, followed by linear transformation using “ScaleData”, dimensionality reduction using “RunPCA” (50 PCs), and clustering using “FindClusters” (resolution 0.5) resulting in 16 clusters. DEGs were identified using the “FindAllMarkers” function with default parameters (min.pct = 0.1, logfc.threshold = 0.25 to focus on genes with a log2‐fold‐change greater than 0.25, and test.use = “wilcox” for the Wilcoxon rank‐sum test). We removed B cell lineage cells from clusters 12 and 14 because these highly expressed *JCHAIN*, resulting in a total of 9267 cells. Next, we performed once more “DietSeurat”, “ScaleData”, “RunPCA” (50 PCs), “FindClusters” (resolution 0.5), resulting in 16 clusters. Again, DEGs were identified using the “FindAllMarkers” function with parameters set as above. Based on the expression of known genes we aggregated the 16 clusters in 3 major populations namely fibroblasts, BECs, and LECs.

### 5.5 Fibroblast, BECs, and LECs Dataset

To annotate the fibroblast dataset, we manually selected from the stromal cell dataset those clusters that highly expressed PDGFRB, DCN, and LUM and were also VWF (CD31) negative. Second, for the BECs dataset, we selected clusters that expressed VWF from the stromal cell dataset. Third, for the LECs dataset, we selected those clusters highly expressing LYVE PROX1 and VWF. After filtering, the three datasets were trimmed using the function “DietSeurat”, followed by linear transformation using “ScaleData”, then the top 2000 variable genes were selected using “FindVariableFeatures” for the dimensionality reduction using “RunPCA” (50 PCs). We calculated the UMAP coordinates (50 PCs) and performed clustering resolution 0.5) using the functions “RunUMAP” and “FindClusters”, respectively. DEGs were identified using the function “FindAllMarkers” with parameters set as above.

### 5.6 Gene Set Enrichment Analysis

Gene Set Enrichment Analysis was performed using the R package gsfischer (https://github.com/sansomlab/gsfisher). We selected positively differentially expressed genes for each cluster with an adjusted *p*‐value < 0.05. Enrichment analysis was performed with gene sets (category: molecular function) with at least three genes, at most 500 genes, and an overrepresentation odds ratio of at least 2.

### 5.7 Integration with Public Datasets

For the integration of our scRNA‐seq dataset with publicly available datasets from Abe et al. [8] and Kapoor et al. [5], we adhered to the standard integration workflow outlined in the Seurat package. We note that Abe et al.’s [8] nine samples were derived from the head and neck of patients tested as cancer‐free, while Kapoor et al. [5] provided three samples, all ethically sourced from adult patients undergoing resection. Initially, each dataset underwent preprocessing, including quality control, normalization, and identification of variable features. Following preprocessing, the “FindIntegrationAnchors” function was employed to identify anchors between the datasets, allowing for the discovery of mutual cell states despite originating from disparate sources. These anchors facilitated the harmonization of the datasets, minimizing batch effects while preserving the intrinsic biological variability. Subsequently, the “IntegrateData” function was utilized to merge the datasets into a single, integrated dataset. This integrated dataset provided a comprehensive view, enabling the comparative analysis of LNSC subsets in human LN tissue from 13 lymph nodes across different two publicly available scRNA‐seq studies and our dataset. Demographic information of each dataset can be found in Supporting Information Table 7.

### 5.8 AddModuleScore Analysis

In our methodology, the “AddModuleScore” function from the Seurat package was utilized to quantify the expression signature of the novel fibroblast subsets identified in our study. For each subset, a list of 10 unique DEGs was curated. These genes were chosen based on their specificity and differential expression levels, ensuring minimal overlap with genes associated with other cell types. This score was then mapped across the identified clusters of the integrated dataset to assess the preservation and distinctiveness of the subsets in the integrated analysis, facilitating a deeper understanding of the LNSC landscape across varying datasets. The resultant module scores were visualized via a heatmap, enabling the identification of cluster‐specific expression patterns and the validation of the novel subsets within the integrated LNSC dataset.

### 5.9 Spatial Transcriptomics Dataset Analysis

The human lymph node spatial dataset was downloaded from the dataset portal provided by 10× Genomics (https://support.10xgenomics.com/spatial‐gene‐expression/datasets/1.1.0/V1_Human_Lymph_Node). The dataset contains 4,035 detected spots, 20,239 median UMI counts per spot, and 5999 median genes per spot. Low‐quality spots corresponding to spots expressing < 1000 genes or > 20% of mitochondrial‐associated reads were filtered out. The filtered count data were analyzed by the Seurat package (version 3.2). The dataset was normalized using the “SCtransform” function [86]. The first 50 PCAs were used for UMAP dimensionality reduction and cell clustering. The resolution of “FindClusters” was set to 0.8, because the resulting clusters were consistent with the mapping of known markers in each area of the sequenced section.

### 5.10 Transfer of Labels to the Spatial Transcriptomics Dataset

We applied the anchor‐based integration workflow introduced in Seurat v3, using the “FindTransferAnchors” function. This approach enables the probabilistic transfer of annotations from a reference dataset (our scRNA‐seq data) to a query dataset (spatial transcriptomics). Anchors, which represent pairwise correspondences between individual cells in each dataset, serve as the basis for transferring information. UsingTransferData’ (weight.reduction = “pcaproject”), these anchors facilitate the projection of reference annotations onto the query dataset. The procedure outputs prediction scores for each spot, providing a probabilistic classification into scRNA‐seq‐derived clusters. The main steps of this process are outlined below. For a detailed description of the methodology, refer to the study by Stuart et al. [30].

### 5.11 Ligand Receptor Analysis with NicheNet

NicheNet analysis was adapted from the vignettes described at https://github.com/saeyslab/nichenetr/blob/master/vignettes/circos.md, https://github.com/saeyslab/nichenetr/blob/master/vignettes/seurat_steps.md. From our dataset, B and T cells were selected based on the SingleR annotation (Table S1). To increase the numbers of B and T cells for this analysis, the lymph node Tabula Sapiens dataset [23] was downloaded from https://datasets.cellxgene.cziscience.com/a1910856‐f7ab‐429d‐b208‐d0da57be6beb.rds. Subsequently, RNAseq data from B and T cells from our dataset were integrated with the data from B and T cells from the Tabula Sapiens dataset [23]. The T and B cells from the two datasets were integrated with the following functions in R from the Seurat package, SelectIntegrationFeatures(), FindIntegrationAnchors(), IntegrateData(). This resulted in a dataset containing 21879 B‐cells and 23198 T‐cells for subsequent receptor–ligand analysis. As NicheNet requires the selection of a population as “receiver” and of the others as “sender”, we performed two NicheNet runs, one with T cells (receiver) and CCL19+ SC, CD34+CXCL14+ SC, HLA‐DR+ SC (senders), and a second one with B cells (receiver) and GLDN+ SC, NR4A1+ BCAM+ SCs, SEPT4+ SC (senders). With the two groupings, stroma–T cell interactions and stroma–B cell interactions, the expressed ligands of each stromal cell subset were determined by intersecting the differentially expressed genes of each subset with the ligand–receptor network (lr_network) retrieved from https://zenodo.org/record/3260758/files/lr_network.rds.

The expressed receptors in the T and B cells were determined using the get_expressed_genes() function and the resulting expressed genes were intersected with the lr_network. To further refine the selection, potential ligands were identified by filtering the lr_network to retain only those interactions where the ligand was present in the expressed ligands list, and the receptor was found in the expressed receptors list. After identifying potential ligands, the analysis proceeded by defining the background gene set for further NicheNet calculations. This step selects genes expressed in the receiver cells (T or B cells) that are also present in the ligand–receptor matrix. Next, we computed the ligand activity using the function predict_ligand_activities(). After the ligand activities were computed, they were ranked by their correlation strength (Pearson correlation coefficient), with the highest‐ranking ligands being the most strongly associated with receptor gene expression in the receiver cells. Following the ranking of ligand activities, the analysis focused on assessing the expression levels of the top‐ranked ligands across different stromal cell subsets. This step involves calculating the average expression of each ligand within specific stromal cell types. The top five ligands for each stromal cell subset were chosen based on their correlation strength with the receptor genes, as indicated by the Pearson correlation coefficient. These top five ligands for each subset were subsequently used to construct the ligand–receptor network and visualized in a heatmap (Figure 4A,B). Complete tables are provided for the inferred ligand–receptor interactions of stromal cells with B cells (Supporting Information Table 5) and T cells (Supporting Information Table 6).

### 5.12 Fluorescent Immunohistochemistry Staining of Frozen Lymph Node Section

One renal lymph node and two hepatic lymph nodes were embedded in an optimal cutting temperature compound (OCT, Fischer Scientific) and directly frozen in liquid nitrogen. The peritoneal lymph node was acquired according to the guidelines of the Declaration of Helsinki and with approval by the Ethics Committee of the Amsterdam UMC, location AMC (protocol code 2016_045; 1 April 2016). The hepatic lymph nodes were obtained from donors during liver transplant procedures performed at the Erasmus MC, University Medical Center Rotterdam, the Netherlands, in accordance with the Medical Ethical Committee (Medisch Ethische Toetsings Commissie; METC) of Erasmus MC (MEC‐2014‐060). Both liver transplant recipients gave written informed consent to use their donor tissue. The lymph nodes were resected along the hepatic artery and portal vein in the porta hepatis from donor livers. The OCT frozen tissue blocks were cut into 7 µm‐thick sections for further analysis. Frozen sections were acetone‐fixed for 10 min at room temperature (RT). After washing with PBS supplemented with 2% newborn calf serum (nbcs, Lonza, cat# DE14‐417EH) (PBS‐nbcs), the sections were blocked for 15 min at RT with 10% normal human serum (Lonza, cat# 14–402E) in PBS‐nbcs to prevent nonspecific binding. Then, sections were incubated with primary antibody diluted in PBS‐nbcs for 30 min at RT. After three washes, sections were incubated with a secondary antibody diluted in PBS‐nbcs for 15 min at RT. These staining steps were repeated to stain in sequence GLDN (rabbit, Invitrogen, cat# PA5‐115286, clone CRG‐L2), PDPN (rat, AngioBio, cat# 11‐009, clone NZ‐1), NEFL (mouse, Invitrogen, cat# MA5‐17135, clone 1H3), CD19 (Biolegend, cat# 302204, clone HIB19), Anti‐Human CD31‐AF488 (Biolegend, Cat# 303110, clone WM59), CD21L (in‐house produced with hybridoma, clone 7D6), and CD34 (Biolegend, cat# 343507, clone 581). Secondary antibodies used were goat‐α‐rat (AF594, Invitrogen, cat# A11007), donkey‐ α‐rabbit (AF647, Invitrogen, cat# A31573), goat‐α‐mouse (AF488, Invitrogen, cat# A11017) and streptavidine‐AF555 (Invitrogen, cat# S32355). When necessary to block the nonspecific binding of the secondary antibody, 2% normal rat serum or 2% normal rabbit serum (produced in‐house, in collaboration with the Amsterdam Animal Research Center (AARC)) was added to the secondary antibody staining. Furthermore, before CD19 staining, the sections were blocked with 20% normal mouse serum (produced in‐house, in collaboration with the AARC) in PBS‐nbcs for 15 min at RT. Lastly, nuclei were counterstained with Sytox Blue (Invitrogen, cat# S11348) and mounted with Mowiol (Sigma Aldrich, cat# 81381). Images were acquired with the Leica TCS SP8 STED 3× confocal microscope (Leica microsystems, IR GmbH) and analyzed with ImageJ [87].

### 5.13 Visualization

Log‐normalized gene expression data was used for visualizations with violin plots (VlnPlot), and UMAP plots (FeaturePlot). Scaled log‐normalized gene expression data was used for visualizations with dot plots (DotPlot) and heatmaps (DoHeatmap). We plotted proportions with barplot (Dittoplot). Prediction scores were displayed with “SpatialFeaturePlot”.

## Author Contributions

Conceptualization: Reina E. Mebius and Lisa G. M. van Baarsen. Methodology: Reina E. Mebius, Lisa G. M. van Baarsen, Perry D. Moerland, and Cristoforo Grasso. Investigation: Cristoforo Grasso, Janna E. G. Roet, Reina E. and Mebius, Lisa G. M. van Baarsen with assistance from Catarina Gago de Graça, Johanna F. Semmelink, Aldo Jongejan, and Ester Remmerswaal. Formal analysis: Cristoforo Grasso with assistance from Perry D. Moerland, Aldo Jongejan, and Michael de Kok. Resources: Ester Remmerswaal. Writing—original draft: Cristoforo Grasso, Reina E. Mebius, and Lisa G. M. van Baarsen. Writing—review & editing: Cristoforo Grasso, Reina E. Mebius, Lisa G. M. van Baarsen, and Perry D. Moerland. Supervision and funding acquisition: Reina E. Mebius and Lisa G. M. van Baarsen.

## Conflicts of Interest

The authors declare no conflicts of interest.

## Supporting information




**Supporting File 1**: eji5978‐sup‐0001‐SuppMat.pdf.

**Supporting File 2**: eji5978‐sup‐0002‐Figures.docx.

**Supporting File 3**: eji5978‐sup‐0003‐TableCaptions.docx.

**Supporting File 4**: This table shows the number of cells per cell type following annotation with SingleR. The frequency provides an overview of the cellular composition in the dataset, including various immune cells, fibroblasts, muscle cells, and others.

**Supporting File 5**: Differential gene expression between the 4 LEC subsets described in this study reported in figure S2d. Columns include p‐value (p_val), average log fold change (avg_log2FC), adjusted p‐value (p_val_adj), and the proportions of cells in each group expressing the gene (pct.1 and pct.2). These data provide insight into gene expression levels and patterns of each cluster.

**Supporting File 6**: Differential gene expression betweenthe 4 BEC subsets described in this study reported in figure S3b. Columns include p‐value (p_val), average log fold change (avg_log2FC), adjusted p‐value (p_val_adj), and the proportions of cells in each group expressing the gene (pct.1 and pct.2). These data provide insight into gene expression levels and patterns of each cluster.

**Supporting File 7**: Differential gene expression between fibroblasts, BECs, and LECs reported in the heatmap in Figure 1f. Columns include p‐value (p_val), average log fold change (avg_log2FC), adjusted p‐value (p_val_adj), and the proportions of cells in each group expressing the gene (pct.1 and pct.2). These data provide insight into gene expression levels and patterns of each cluster.

**Supporting File 8**: Differential gene expression between the 10 LN fibroblast subsets described in this study (related to Figure 2c). Columns include p‐value (p_val), average log fold change (avg_log2FC), adjusted p‐value (p_val_adj), and the proportions of cells in each group expressing the gene (pct.1 and pct.2). These data provide insight into gene expression levels and patterns of each subset.

**Supporting File 9**: Differential gene expression related to the integrated dataset in Figure 2f. This table displays differentially expressed genes for each identified cluster. Columns include p‐value (p_val), average log fold change (avg_log2FC), and adjusted p‐value (p_val_adj) reflecting expression differences and statistical significance. pct.1 and pct.2 indicate the proportion of cells in each group expressing the gene.

**Supporting File 10**: Differential gene expression of the spatial dataset related to Figure 3b. This table displays differentially expressed genes between identified spatial areas. Columns include p‐value (p_val), average log fold change (avg_log2FC), and adjusted p‐value (p_val_adj) reflecting expression differences and statistical significance. pct.1 and pct.2 indicate the proportion of cells in each group expressing the gene.

**Supporting File 11**: Ligand‐receptor interactions between SEPT4+SC, NR4A1+ BCAM+ SC, GLDN+ and B‐cells (related to Figure 4a). This table summarizes key ligand‐receptor pairs identified by NicheNet analysis, focusing on interactions between SEPT4+SC, NR4A1+ BCAM+ SC subsets, and B‐cells. Columns include the ligand, receptor, interaction strength (weight), receptor‐expressing cell type (receptor_type), and ligand classification (ligand_type), highlighting signaling pathways within the B‐cell context.

**Supporting File 12**: Ligand‐receptor interactions between HLA‐DR+SC, CCL19+SC, CD34+CXCL14+SC and T‐cell (related to Figure 4b) This table summarizes key ligand‐receptor pairs identified by NicheNet analysis, illustrating signaling interactions involving T‐cells. Columns show the ligand, receptor, interaction strength (weight), receptor‐expressing cell type (receptor_type), and ligand classification (ligand_type).

**Supporting File 13**: This table details the characteristics of LN donors used in the various datasets (Kapoor et al., Abe et al., Grasso et al., and the public 10x Visium data) described in this study. Columns include sample type, age, sex, confirmed diagnosis, and protocol used, providing context on the samples' origin and preparation for gene expression analysis.

## Data Availability

The data that support the findings of this study are openly available in scRNA‐seq data have been deposited and are publicly available in the GEO database at https://www.ncbi.nlm.nih.gov/geo/query/acc.cgi?&acc=GSE261747, reference number GSE261747. The integrated dataset containing the data of Grasso et al., Abe et al., and Kapoor et al. can be found in Zenodo at https://zenodo.org/uploads/14011548 reference number 14011548. The analysis was performed with R version 4.0.1 (2020‐06‐06) running under Windows 10 (x64), and the main R packages used were Seurat (3.2), SingleR (3.12), and NicheNet (1.1.0).
