# Supplementary material for: Identification and Mapping of Human Lymph Node Stromal Cell Subsets by Combining Single‐Cell RNA Sequencing with Spatial Transcriptomics
Source: Eur J Immunol. 2025 Jun 11;55(6):e51218. doi: 10.1002/eji.202451218 (PMC12154172; doi:10.1002/eji.202451218)
Supplement: Supplementary file 2 — Supporting File 2: eji5978‐sup‐0002‐Figures.docx. [file EJI-55-e51218-s012.docx]

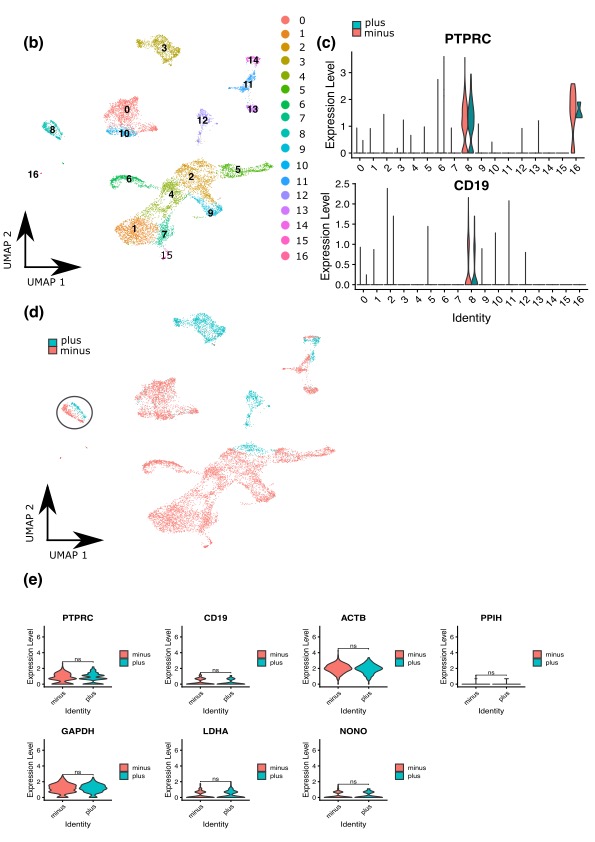


**Figure S1. Sorting strategy and 10X Genomics quality control**

**a.** Fluorescence-activated cell sorting (FACS) gating strategy to isolate viable cells specifically excluding CD45+ and CD235a+ cells. Gating ensured a balance between HLA-DR+ and HLA-DR- LNSCs, including double negative (DN), fibroblastic reticular cells (FRC), lymphatic endothelial cells (LEC), and blood endothelial cells (BEC). Additionally, B cells from the same sorting experiment were spiked into both 10X Genomics wells to control for batch effects. **b.** UMAP clustering visualizing all sorted cells and showing the distribution of various clusters corresponding to different LNSC subsets and spiked B cells **c.** Violin plot showing the Z-score gene expression levels of known B-cell markers *CD19* and *PTPRC* (*CD45*) within the clusters. **d.** UMAP clustering visualizing the two 10X Genomics wells (plus means sorted HLA-DR+ LNSCs and minus means sorted HLA-DR- LNSCs) and marking the spiked B-cell cluster with a circle. **e.** Violin plots comparing the Z-score normalized expression levels of housekeeping genes (ACTB, PPIH, GAPDH, LDHA, NONO) and B-cell markers (CD19, PTPRC) between the two wells. The statistical comparison was performed using the Wilcoxon signed-rank test, with n.s. indicating non-significant differences (P > 0.05).

**Figure S2. Identification of four lymphatic endothelial cell (LEC) clusters within the lymph node**

**a.** UMAP (Uniform Manifold Approximation and Projection) plot displaying the clustering of lymphatic endothelial cells (LECs) into four distinct subsets. Each LEC cluster is represented by a different colour. **b.** Bar plot showing the absolute number of cells within each of the four LEC clusters. The bars are color-coded to correspond with the clusters identified in the UMAP plot, providing a quantitative breakdown of the cluster sizes. **c.** Violin plots illustrating the Z-score normalized expression levels of key marker genes for each LEC cluster. These plots display the variation in expression of the top marker genes. **d.** Heatmap depicting the expression levels of the top 50 marker genes across the four LEC clusters. Key marker genes for each LEC subset are highlighted on the left. The heatmap colour scale represents the relative expression levels (Z-scores).

**Figure S3. Identification of four blood endothelial cell (BEC) clusters within the lymph node**

**a.** UMAP plot displaying the clustering of blood endothelial cells into four distinct subpopulations within the lymph node. Each cluster is color-coded, reflecting its unique gene expression profile and allowing for clear visualization of the BEC subsets. **b.** Bar plot indicating the absolute number of cells in each of the four BEC clusters. The colours in the bar plot correspond to the clusters identified in the UMAP plot. **c.** Violin plots showing the Z-score normalized expression levels of the top marker genes for each BEC cluster. **d.** Heatmap depicting the expression levels of the top 50 marker genes across the four BEC clusters. Key marker genes for each BEC subset are indicated on the left of the heatmap. The color scale (Z-scores) reflects the relative expression levels.

**Figure S4. Comparative analysis of stromal cell cluster co-occurrence across integrated datasets**

**a.** Stacked bar plot showing the proportional distribution of the cells within the integrated dataset. Each bar represents a fibroblast cluster from the integrated dataset (y-axis), with colour segments indicating the contribution of each dataset (Grasso et al, Abe et al., Kapoor et al.) to the respective cluster. The x-axis represents the fraction of cells within each dataset, ranging from 0.0 to 1.0, with 1.0 indicating 100% of cells within a dataset. **b.** Heatmap displaying the AddModuleScore results for the lymph node stromal cell subsets identified within the integrated datasets (as shown in Figure 2f). The average module scores for each lymph node (LN) fibroblast subset (on the x-axis) are plotted against the integrated clusters (on the y-axis). Warmer colours in the heatmap represent higher module scores, indicating a stronger association of specific fibroblast subsets with certain clusters. **c.** Refined analysis of Cluster 1 following re-clustering within the integrated dataset. The UMAP plot illustrates the re-clustered subsets. **d.** Heatmap displaying the module score results for Cluster 1 following re-clustering from the integrated dataset


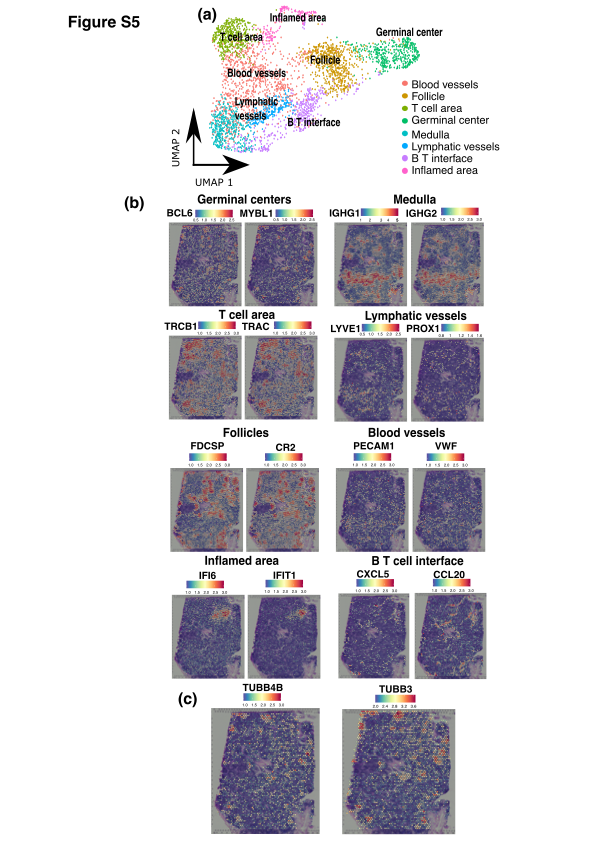


**Figure S5. Annotation of main cellular regions in a human lymph node using spatial transcriptomics**

**a.** UMAP plot displaying the distinct areas of the lymph node identified by unsupervised clustering of spatial transcriptomics data. Each cluster represents a specific region of the lymph node, and clusters were manually annotated based on differentially expressed genes (DEGs), as shown in Figure 3b. **b.** Spatial plots visualizing the expression levels of key marker genes, such as BCL6 and TRBC1, which are highly expressed in specific lymph node areas (e.g., germinal centers and medulla). **c.** Spatial visualization of marker genes, including TUBB4B and TUBB, known to be expressed by neurons. Z-scores indicate the expression levels, with blue representing low expression (Z-score = 0) and red representing high expression (Z-score = max).

**Figure S6**: **UMAP visualization of fibroblast subsets and gene expression in the Grasso et al. dataset and the integrated dataset.** **a.** UMAP plot showing the identified lymph node stromal cell (LNSC) subsets in the Grasso et al. dataset. Subsets include GLDN+ SC, NR4A1+BCAM+ SC, Pericytes, CCL19+ SC, DES+ SC, CCL21+ SC, HLA-DR+ SC, LAMP5+ SC, SEPT4+ SC, and CD34+CXCL14+ SC. Moving to the right, feature plot showing the expression of PI16 and CD34 across cells is depicted. The color scale (Z-scores) reflects the relative expression levels.

**b.** UMAP plot showing the integrated dataset. The plot highlights the CD34+CXCL14+ SC population as well as other major cell types such as keratinocytes and B cells. Moving to the right, feature plot showing the expression of PI16 and CD34 across cells is depicted. The color scale (Z-scores) reflects the relative expression levels.

**Figure S7. NEFL microscopy in human lymph node and brain sections**

**a.** Fluorescent labelling of various cellular markers in a section of human lymph node tissue. The first panel presents the complete lymph node section (acquired at 40X magnification), showing the spatial localization of NEFL (green), GLDN (magenta), PDPN (yellow), and CD19 (cyan). Successive panels provide individual views of each marker's staining, moving from left to right. The final panel is a composite image, merging all markers and including Sytox Blue to label nuclei. Scale bars indicate 20 µm for individual marker panels and their magnified inserts, and 100 µm for the full tissue section. **b.** Immunofluorescence staining of NEFL (green) on a section of human brain tissue, acquired at 40X magnification. The left panel shows NEFL staining using an anti-human NEFL antibody, while the right panel displays an isotype control for comparison. Scale bars represent 100 µm.
