# Supplementary material for: Identification and Mapping of Human Lymph Node Stromal Cell Subsets by Combining Single‐Cell RNA Sequencing with Spatial Transcriptomics
Source: Eur J Immunol. 2025 Jun 11;55(6):e51218. doi: 10.1002/eji.202451218 (PMC12154172; doi:10.1002/eji.202451218)
Supplement: Supplementary file 3 — Supporting File 3: eji5978‐sup‐0003‐TableCaptions.docx. [file EJI-55-e51218-s006.docx]

**Table legends**

**Table 1.** Differential gene expression between fibroblasts, BECs, and LECs reported in the heatmap in Figure 1f. Columns include p-value (p_val), average log fold change (avg_log2FC), adjusted p-value (p_val_adj), and the proportions of cells in each group expressing the gene (pct.1 and pct.2). These data provide insight into gene expression levels and patterns of each cluster.

**Table 2.** Differential gene expression between the 10 LN fibroblast subsets described in this study (related to Figure 2c).. Columns include p-value (p_val), average log fold change (avg_log2FC), adjusted p-value (p_val_adj), and the proportions of cells in each group expressing the gene (pct.1 and pct.2). These data provide insight into gene expression levels and patterns of each subset.

**Table 3.** Differential gene expression related to the integrated dataset in Figure 2f. This table displays differentially expressed genes for each identified cluster. Columns include p-value (p_val), average log fold change (avg_log2FC), and adjusted p-value (p_val_adj) reflecting expression differences and statistical significance. pct.1 and pct.2 indicate the proportion of cells in each group expressing the gene.

**Table 4.** Differential gene expression of the spatial dataset related to Figure 3b. This table displays differentially expressed genes between identified spatial areas. Columns include p-value (p_val), average log fold change (avg_log2FC), and adjusted p-value (p_val_adj) reflecting expression differences and statistical significance. pct.1 and pct.2 indicate the proportion of cells in each group expressing the gene.

**Table 5.** Ligand-receptor interactions between SEPT4+SC, NR4A1+ BCAM+ SC, GLDN+ and B-cells (related to Figure 4a). This table summarizes key ligand-receptor pairs identified by NicheNet analysis, focusing on interactions between SEPT4+SC, NR4A1+ BCAM+ SC subsets, and B-cells. Columns include the ligand, receptor, interaction strength (weight), receptor-expressing cell type (receptor_type), and ligand classification (ligand_type), highlighting signaling pathways within the B-cell context.

**Table 6.** Ligand-receptor interactions between HLA-DR+SC, CCL19+SC, CD34+CXCL14+SC and T-cell (related to Figure 4b) This table summarizes key ligand-receptor pairs identified by NicheNet analysis, illustrating signaling interactions involving T-cells. Columns show the ligand, receptor, interaction strength (weight), receptor-expressing cell type (receptor_type), and ligand classification (ligand_type).

**Table 7.** This table details the characteristics of LN donors used in the various datasets (Kapoor et al., Abe et al., Grasso et al., and the public 10x Visium data) described in this study. Columns include sample type, age, sex, confirmed diagnosis, and protocol used, providing context on the samples' origin and preparation for gene expression analysis.

**Table S1.** This table shows the number of cells per cell type following annotation with SingleR. The frequency provides an overview of the cellular composition in the dataset, including various immune cells, fibroblasts, muscle cells, and others.

**Table S2.** Differential gene expression between the 4 LEC subsets described in this study reported in figure S2d. Columns include p-value (p_val), average log fold change (avg_log2FC), adjusted p-value (p_val_adj), and the proportions of cells in each group expressing the gene (pct.1 and pct.2). These data provide insight into gene expression levels and patterns of each cluster.

**Table S3.** Differential gene expression betweenthe 4 BEC subsets described in this study reported in figure S3b. Columns include p-value (p_val), average log fold change (avg_log2FC), adjusted p-value (p_val_adj), and the proportions of cells in each group expressing the gene (pct.1 and pct.2). These data provide insight into gene expression levels and patterns of each cluster.
